# Cucurbitacin D induces cell cycle arrest and apoptosis by inhibiting STAT3 and NF-κB signaling in doxorubicin-resistant human breast carcinoma (MCF7/ADR) cells

**DOI:** 10.1007/s11010-015-2509-9

**Published:** 2015-07-14

**Authors:** Jin Mo Ku, Soon Re Kim, Se Hyang Hong, Han-Seok Choi, Hye Sook Seo, Yong Cheol Shin, Seong-Gyu Ko

**Affiliations:** Laboratory of Clinical Biology and Pharmacogenomics, Department of Preventive Medicine, College of Oriental Medicine, Kyung Hee University, Seoul, 130-701 Republic of Korea

**Keywords:** Breast cancer, Cucurbitacin D, Doxorubicin, Multidrug resistance, MCF7 cell, MCF7/ADR cell

## Abstract

Breast cancer is the most common cancer for women and is a major cause of mortality in women. Doxorubicin is a generally used chemotherapy drug for breast cancer. However, multidrug resistance of breast cancer interferes with the chemotherapy. We examined whether cucurbitacin D affects doxorubicin resistance of MCF7/ADR breast cancer cells. Cell viability was measured by MTT assay. Levels of p-STAT3, p-NF-κB, IκB, and caspases were measured by Western blot analysis. Nuclear staining of Stat3 and NF-κB was measured by immunocytochemistry. STAT3 and NF-κB transcriptional activity was detected by STAT3 and NF-κB luciferase reporter gene assays. Analysis of cell cycle arrest was performed by flow cytometry. Induction of apoptosis by cucurbitacin D was measured by Annexin V-FITC/propidium iodide assay. More than 90 % of MCF7/ADR cells lived upon treatment with doxorubicin for 24 h. However, upon treatment with cucurbitacin D, cell death was more than 60 %. Co-administration of cucurbitacin D and doxorubicin induced apoptosis, and G2/M cell cycle arrest, and inhibited upregulated Stat3 by doxorubicin on MCF7/ADR cells. Additionally, cucurbitacin D led to an increase in the IκBα level in the cytosol and a decrease in the p-NF-κB level in the nucleus. Finally, cucurbitacin D inhibited translocation of Stat3 and NF-κB and decreased transcriptional activity in the nucleus. Cucurbitacin D decreases cell proliferation and induces apoptosis by inhibiting Stat3 and NF-κB signaling in doxorubicin-resistant breast cancer cells. Cucurbitacin D could be used as a useful compound to treat adriamycin-resistant patients.

## Introduction

Breast cancer is the most common cancer for women and is a major cause of mortality in women [1]. With the development of breast cancer therapy, patient survival rates have greatly improved [2]. Doxorubicin is an anthracycline antibiotic. Anthracycline antibiotic is one of the natural product daunomycin and works by intercalating DNA. It is widely used in the treatments of several cancers [3]. Therefore, doxorubicin is a very important part of a breast cancer therapy regimen and is a generally used agent [4, 5]. However, doxorubicin drug resistance appears in nearly 50 % of treated patients [6]. And doxorubicin induces an upregulation of activated Stat3 and NF-κB activation [7, 8].

Stat3 is a predictive marker of drug resistance [9]. Stat3 was significantly overexpressed in doxorubicin-resistant cells. Thus, Stat3 activation is effective for tumor cell evasion of cancer therapy.

The NF-κB family contains five members: RelA/p65, RelB, c-Rel, p50 (NF-κB1), and p52 (NF-κB2). NF-κB is a dimer composed of p65 and p50. In the cytosol, NF-κB is inactive through interactions with the inhibitor of NF-κB proteins. Exposure to a variety of stimuli leads to phosphorylation of IKKα, IKKβ, and NEMO. Phosphorylated IκBα is ubiquitinated and degraded by the proteasome; then, NF-κB p65 protein is phosphorylated by IKK, and then translocated into the nucleus [10, 11]. Activation of NF-κB promotes proliferation, inflammation, and tumorigenesis in cancer [12, 13].

Signal transducers and activators of transcription (Stat) proteins comprise a seven-member family of transcription factors (Stats 1, 2, 3, 4, 5a, 5b, and 6). Stats transduces signal from extracellular stimulus to the transcription of target genes. Among the Stats, Stat3 is the most widely related with tumor development [14]. Stat3 is activated by phosphorylation of a single tyrosine residue located at position 705 [15]. Stat3 activation in tumor cells is associated with cell proliferation, cell survival, invasion, angiogenesis, and metastasis. Recently, the Stat3 signaling pathway has been shown to confer resistance to chemotherapy-induced apoptosis in human tumors [16–19].

Cucurbitacins are a group of triterpenoids isolated from plant families Cucurbitaceae and Cruciferae. Cucurbitacins have anti-inflammatory activity and anti-cancer effects on various tumors [20]. Cucurbitacins B, D, E, I, F, O, P, and Q are known to suppress proliferation of tumor cells through inhibition of STAT3 phosphorylation [21]. Recently, it was reported that cucurbitacin D from *Trichosanthes kirilowii* has the ability to induce apoptosis in cancer. Cucurbitacin D impedes Stat3 and NF-κB nuclear translocation. Cucurbitacin suppresses cell growth and produces apoptosis in various cancer cell lines [22, 23]. However, the effect of cucurbitacin D has not been investigated in breast cancer cells.

Stat3 and NF-κB signaling pathways play a critical role in cancer cells. Additionally, activated p-NF-κB and p-Stat3 interaction increased intercellular adhesion levels, migration, and invasion [24, 25]. Thus, Stat3 and NF-κB decreases are very important in cancer therapy. It is known that cucurbitacin D suppresses STAT3 and NF-κB activity inhibiting their nuclear translocation and transcriptional activity [22, 26]. In the present study, we examined whether cucurbitacin D affected MCF7/ADR breast cancer cells.

## Materials and methods

### Reagents

Cucurbitacin D was purchased from Extrasynthese (Genay Cedex, France). DMSO and MTT were purchased from Sigma-Aldrich (St. Louis, MO, USA). Propidium iodide (PI) was purchased from Invitrogen (Carlsbad, CA, USA). Annexin V, Alexa Fluor 488 conjugate was obtained from Life Technologies (Eugene, OR, USA). The antibodies against cleaved caspase-8, -3, p-STAT3 (Try705), p-IκB (Ser32/36), p-NF-κB p65 (Ser536), pro-caspase-3, and total STAT3 were obtained from Cell Signaling (Danvers, MA, USA). The antibodies against IKK, PARP/p85, p-IKK, and total NF-κB were obtained from Santa Cruz Biotechnology (Dallas, Texas, USA). IκB antibody was obtained from Millipore. Tubulin antibody was obtained from Sigma-Aldrich (St. Louis, MO, USA). ABC kit and diaminobenzidine tetrachloride (DAB) were obtained from Vector (Burlingame, CA, USA).

### Cell culture

MCF7 is a breast cancer cell line. MCF7/ADR cells have been widely used as a multidrug-resistant breast cancer cell model. MCF7/ADR cells and MCF7 breast cancer cells obtained from American-Type Culture Collection were maintained in RPMI1640 supplemented with 10 % heat-inactivated fetal bovine serum (Invitrogen, Carlsbad, CA, USA) and 100 U/mL antibiotic–antimycotic (Invitrogen). Cells were maintained at 37 °C in a humidified incubator with 5 % CO_2_.

### Cell viability assay

Cell viability was measured using the MTT assay. Cells were plated in 96-well flat bottom tissue culture plates at a density of 3 × 10^3^ cells/well and incubated for 24 h. Cells were cultured for an additional 24 h with cucurbitacin D (0.125–16 μg/mL) or doxorubicin (0.04–25 μM). After incubation, MTT reagents (0.5 mg/mL) were added to each well, and the plates were incubated in the dark at 37 °C for another 2 h. The medium was removed, the formazan was dissolved in DMSO, and the optical density was measured at 570 nm using an ELISA plate reader.

### Nuclear and cytoplasmic fractionation

Adherent cells were washed twice with phosphate-buffered saline (PBS), and then collected by scraping and pelleted by centrifugation. Cells were then transferred into a prechilled microcentrifuge tube and gently resuspended in 150 μL hypotonic buffer (20 mM Tris–HCl, pH 7.4, 10 mM NaCl, 3 mM) by pipetting up and down several times. Cells were incubated on ice for 15 min, and the homogenates were centrifuged for 10 min at 3000 rpm at 4 °C. The supernatants, which contained the cytoplasmic fraction, were transferred and saved. Nuclear pellets were resuspended in 500 μL complete cell extraction buffer (100 mM Tris pH 7.4, 2 mM sodium orthovanadate, 100 mM NaCl, 1 % Triton X-100, 1 mM EDTA, 10 % glycerol, 1 mM EGTA, 0.1 % SDS, 1 mM sodium fluoride, 0.5 % deoxycholate, 20 mM sodium pyrophosphate tetrabasic, 1 mM PMSF, protease inhibitor, and dithiothreitol), and incubated on ice for 30 min with vortexing at 10 min intervals. The homogenates were centrifuged for 30 min at 14,000 rpm at 4 °C. The supernatants (nuclear fraction) were transferred to a clean microcentrifuge tube, and then aliquoted and stored at −80 °C for further assay.

### Western blot analysis

Cells were harvested, incubated in one volume of lysis buffer (50 mM Tris–Cl pH 7.4, 1 % NP-40, 0.25 % sodium deoxycholate, 0.1 % SDS, 150 mM NaCl, 1 mM EDTA, and protease inhibitor) for 20 min and centrifuged at 13,000 rpm at 4 °C for 20 min. Aliquots containing 20 μg of protein were separated by SDS-polyacrylamide gel electrophoresis using 8–12 % gels and transferred to nitrocellulose membranes (Protran nitrocellulose membrane, Whatman, UK). Membranes were blocked with 5 % nonfat milk and probed with specific primary antibodies. Membranes were then incubated with horseradish peroxidase-conjugated secondary IgG antibody (Calbiochem, San Diego, CA, USA) and visualized using the enhanced chemiluminescence detection system (Amersham ECL kit, Amersham Pharmacia Biotech, Inc., Piscataway, NJ, USA).

### Flow cytometric analysis

Flow cytometry was used to analyze cell cycle distribution and apoptosis. Cells were seeded in 60-mm dishes. After 24 h, cells were cultured for an additional 24 h in the absence (control) or presence of doxorubicin (1 μM) and/or cucurbitacin D (0.5 or 2 μg/mL). Trypsinized cells were washed with PBS and fixed in 95 % ethanol containing 0.5 % Tween-20 overnight at −20 °C. After washing with PBS, cells were then incubated with 1 U/mL of RNase A and 10 μg/mL of PI for 30 min at room temperature in the dark. The DNA content in each cell nucleus was determined by a FACScalibur flow cytometer (Becton–Dickinson, San Jose, CA, USA), and the cell cycle was analyzed using ModFit LT V2.0 software.

### Annexin V-FITC apoptosis assay

Cells were cultured in 60-mm dishes. After 24 h, cells were cultured for an additional 24 h in the absence (control) or presence of doxorubicin (1 μM) and/or cucurbitacin D (0.5 or 2 μg/mL). Apoptosis assay was performed with an Annexin V-FITC/PI double staining apoptosis detection kit using a flow cytometer following the manufacturer’s instruction.

### Transfection and luciferase assay

For the assay, MCF7/ADR cells were plated and allowed to attach by overnight incubation. The next day, the cells were transfected with control Renilla RNA (Qiagen, Venlo, Netherlands) and STAT3 CA RNA (Santa Cruz, CA, USA) in the presence of a STAT3-luciferase reporter using Lipofectamine 2000 (Invitrogen, Carlsbad, CA, USA). The cells were then treated with doxorubicin or cucurbitacin D for 6 h. Luciferase assays were performed using a dual-luciferase assay kit (Promega, Madison, WI, USA) according to the manufacturer’s instructions. Briefly, cells were lysed using a passive lysis buffer. Cell lysates were then centrifuged, and the supernatant was saved for analysis. Finally, luciferase activities were determined using a luminometer (BMG Labtech, Ortenberg, Germany).

### Immunocytochemical analysis

MCF7/ADR cells were inoculated at a density of 3 × 10^4^ cells per well in eight-well chamber slides (BD Falcon, Bedford, MA). The next day, the cells were treated with cucurbitacin D or doxorubicin for 6 h. The cells were fixed with 4 % paraformaldehyde for 30 min and treated with 3 % hydrogen peroxide (H_2_O_2_) in methanol for 20 min to quench the endogenous peroxidase activity. The cells were washed with PBS, blocked with 5 % BSA in PBS for 1 h and incubated with the anti-NF-κB and anti-STAT3 primary antibody (1:100 dilution) overnight at 4 °C. After washing with PBS, the cells were incubated with the anti-rabbit biotin-conjugated secondary antibody for 1 h at room temperature. Then, the cells were treated with Vectastain ABC reagent (Vector Laboratories, Inc., Burlingame, CA, USA) for 30 min at 4 °C and stained with DAB and hematoxylin. The cells were mounted with mounting medium and subsequently analyzed by microscopy.

### Statistical analysis

The results of all experiments were expressed as the mean ± standard deviation (SD) of at least three separate tests. A Student’s *t*-test was used for single-variable comparisons, and a *p* value <0.05 was considered statistically significant.

## Results

### Effect of doxorubicin, cucurbitacin D, and doxorubicin with cucurbitacin D on MCF7 and MCF7/ADR cell viability

We investigated whether doxorubicin, cucurbitacin D, and doxorubicin with cucurbitacin D affected the cell viability of MCF7 and MCF7/ADR cells.

**Fig. 1:**
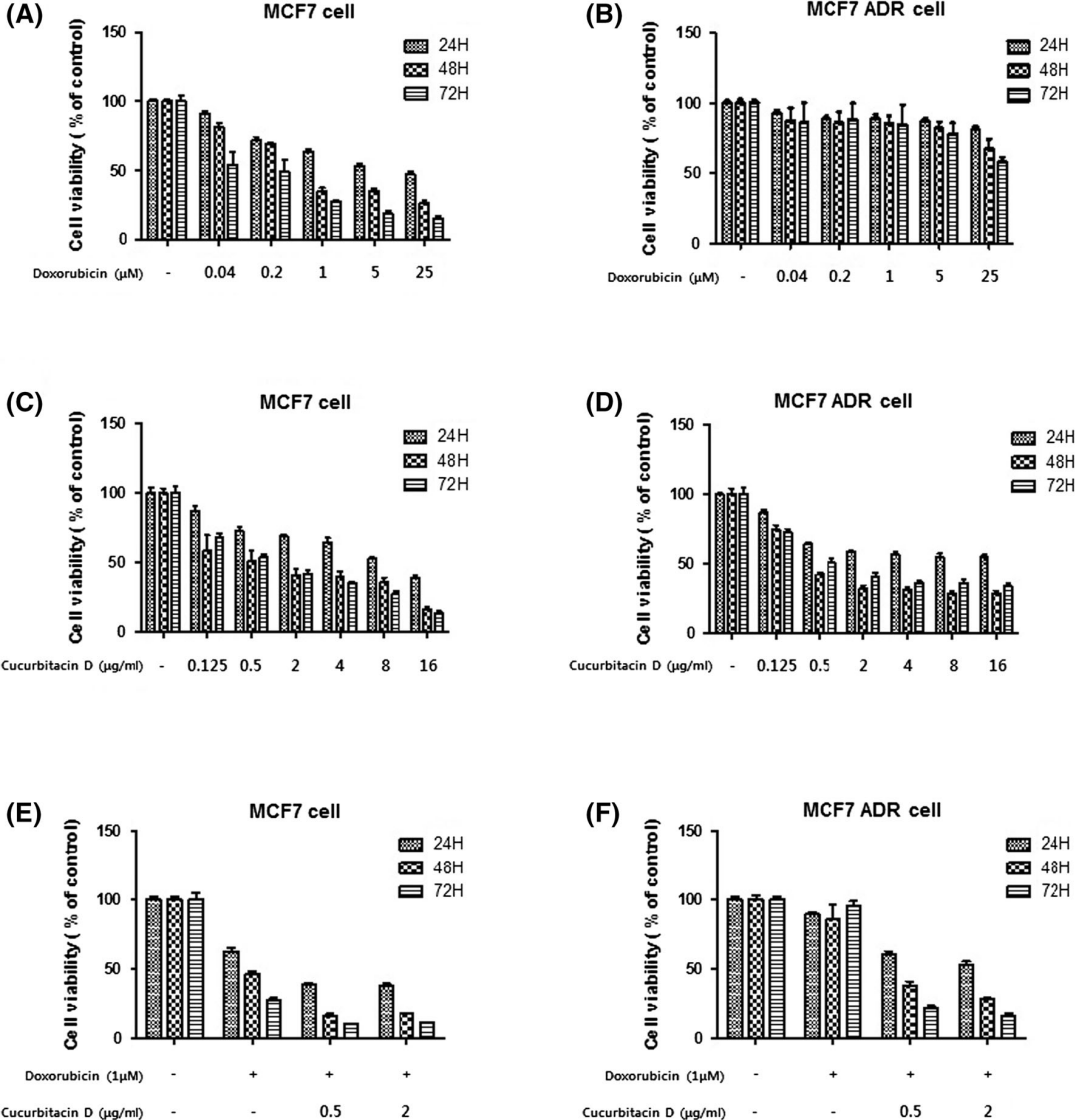
Effect of doxorubicin, cucurbitacin D, and doxorubicin with cucurbitacin D on MCF7 and MCF7/ADR cell viability. MCF7 and MCF7/ADR cells were treated with different concentrations of doxorubicin, cucurbitacin D, cucurbitacin D (0.5, 2 μg/mL), and doxorubicin (1 μM) for 24, 48, and 72 h. Cell viability was then measured using the MTT assay. Each value represents the mean ± SD. All data are *p* < 0.0001 by Student *t*-test

For that purpose, both MCF7 and MCF7/ADR cells were treated with different concentrations of doxorubicin (0.04, 0.2, 1, 5, and 25 μM) for 24–72 h. Cell viability was then measured by MTT assay. We found that doxorubicin significantly suppressed cell growth in a dose- and time-dependent manner in MCF7. However, doxorubicin failed to decrease cell viability in MCF7/ADR cells (Fig. 1a, b). Both MCF7 and MCF7/ADR cells were treated with different concentrations of cucurbitacin D (0.125, 0.5, 2, 4, 8, and 16 μg/mL) for 24–72 h. We found that cucurbitacin D significantly suppressed cell growth in both MCF7 and MCF7/ADR in a dose- and time-dependent manner (Fig. 1c, d). We co-treated MCF7 and MCF7/ADR cells with doxorubicin (1 μM) and cucurbitacin D (0.5, 2 μg/mL) to investigate whether cucurbitacin D overcomes doxorubicin resistance. We found that cucurbitacin D and doxorubicin significantly suppressed cell growth in MCF7 cells, and cucurbitacin D reversed the doxorubicin-resistant cell growth of the MCF7/ADR cell line (Fig. 1e, f).

### Cucurbitacin D suppresses Stat3 expression in MCF7/ADR cells

**Fig. 2:**
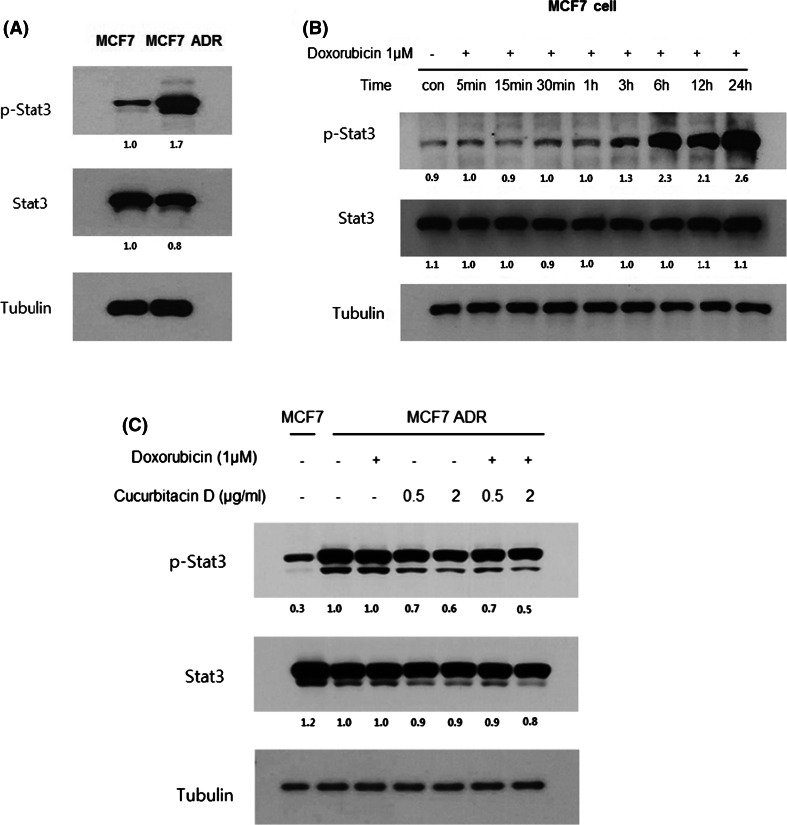
Cucurbitacin D suppresses p-Stat3 expression in MCF7/ADR Cells. Constitutive activation of Stat3 was detected in MCF7/ADR cells. p-STAT3 was strongly expressed in MCF7/ADR cells. **a** Doxorubicin increases constitutive STAT3 phosphorylation in a time-dependent manner in MCF7 cells. **b** Cucurbitacin D inhibits p-Stat3 expression in MCF7/ADR cells. **c** Whole-cell lysates were analyzed by Western blot with anti-pStat3, anti-Stat3, and anti-tubulin antibodies

To determine the relationship between Stat3 and doxorubicin resistance in human breast cancer cells, we analyzed the expression of p-STAT3 and Stat3 in MCF7/ADR cells and MCF7 cells. We found that p-Stat3 expression was significantly overexpressed in MCF7/ADR cells than in the MCF7 cell line (Fig. 2a). Next, we determined whether doxorubicin increased p-Stat3 expression in MCF7 cells. We found that doxorubicin alone increased p-STAT3 level in a time-dependent manner (Fig. 2b).

To further characterize the Stat3-inhibitory effect of cucurbitacin D and doxorubicin in MCF7/ADR cells, we found that cucurbitacin D decreased p-STAT3 level in the absence and presence of doxorubicin, suggesting that cucurbitacin D inhibits STAT3 signaling in MCF7/ADR cells (Fig. 2c).

### Cucurbitacin D inhibits the NF-κB signaling pathway in MCF7/ADR cells

**Fig. 3:**
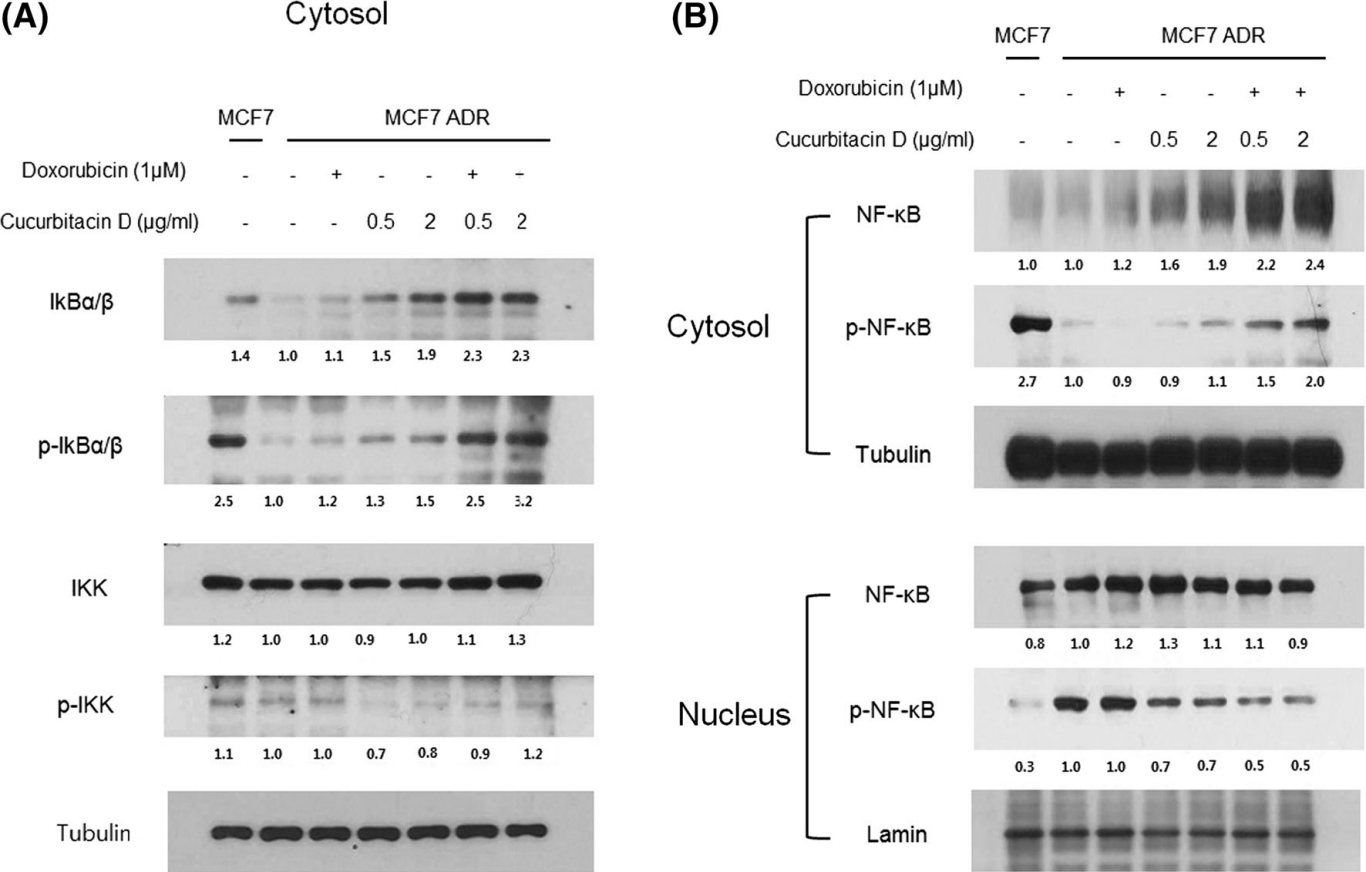
Cucurbitacin D inhibits NF-κB signaling in MCF7/ADR cells. MCF7/ADR cells were treated with cucurbitacin D (0.5, 2 μg/mL) in the presence and absence of doxorubicin (1 μM). Nuclear and cytosolic extracts of cultured cells were then prepared and analyzed by Western blot to measure NF-κB levels

We investigated whether cucurbitacin D inhibits NF-κB signaling in MCF7/ADR cells. For that purpose, we treated MCF7/ADR cells with cucurbitacin D (0.5, 2 μg/mL) and/or doxorubicin (1 μM), and we prepared nuclear and cytosolic fractions of the treated cells subjected them to Western blot analysis. We found that cucurbitacin D increased IκB and NF-κB expression levels in the cytosol and decreased p-NF-κB level in the nucleus (Fig. 3a, b).

### Cucurbitacin D suppresses Stat3 and NF-κB translocation in MCF7/ADR cells

**Fig. 4:**
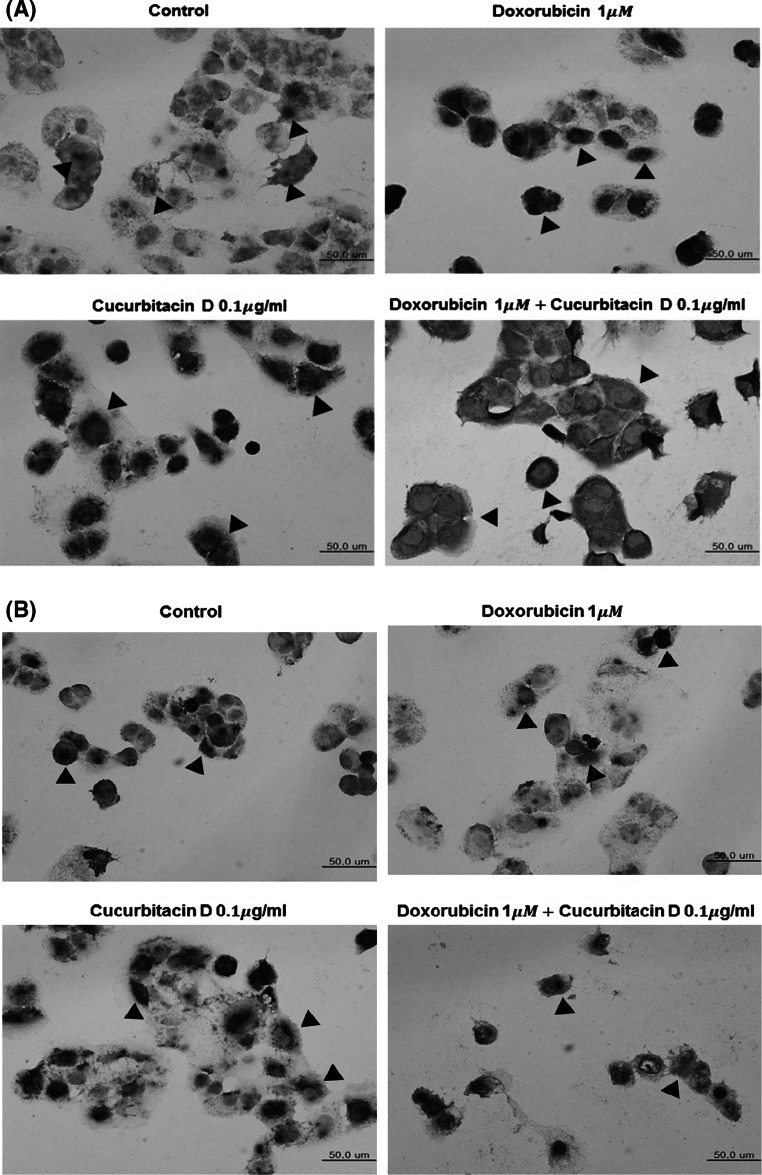
Cucurbitacin D inhibits nuclear translocation of Stat3 and NF-κB in MCF7/ADR cells. MCF7/ADR cells were treated with cucurbitacin D (0.1 μg/mL) in the presence and absence of doxorubicin (1 μM), and then submitted to immunocytochemistry for the detection of nuclear NF-κB (**a**) and Stat3 (**b**)

To confirm that cucurbitacin D inhibits nuclear translocation of Stat3 and NF-κB, we performed immunocytochemistry on MCF7/ADR cells. We found that cucurbitacin D marked cytosol presence of STAT3 and NF-κB and decreased nuclear staining of NF-κB (Fig. 4a) and Stat3 (Fig. 4b) in MCF7/ADR cells. This suggests that cucurbitacin D inhibits nuclear translocation of Stat3 and NF-κB.

### Cucurbitacin D suppresses Stat3 and NF-κB transcriptional activity

**Fig. 5:**
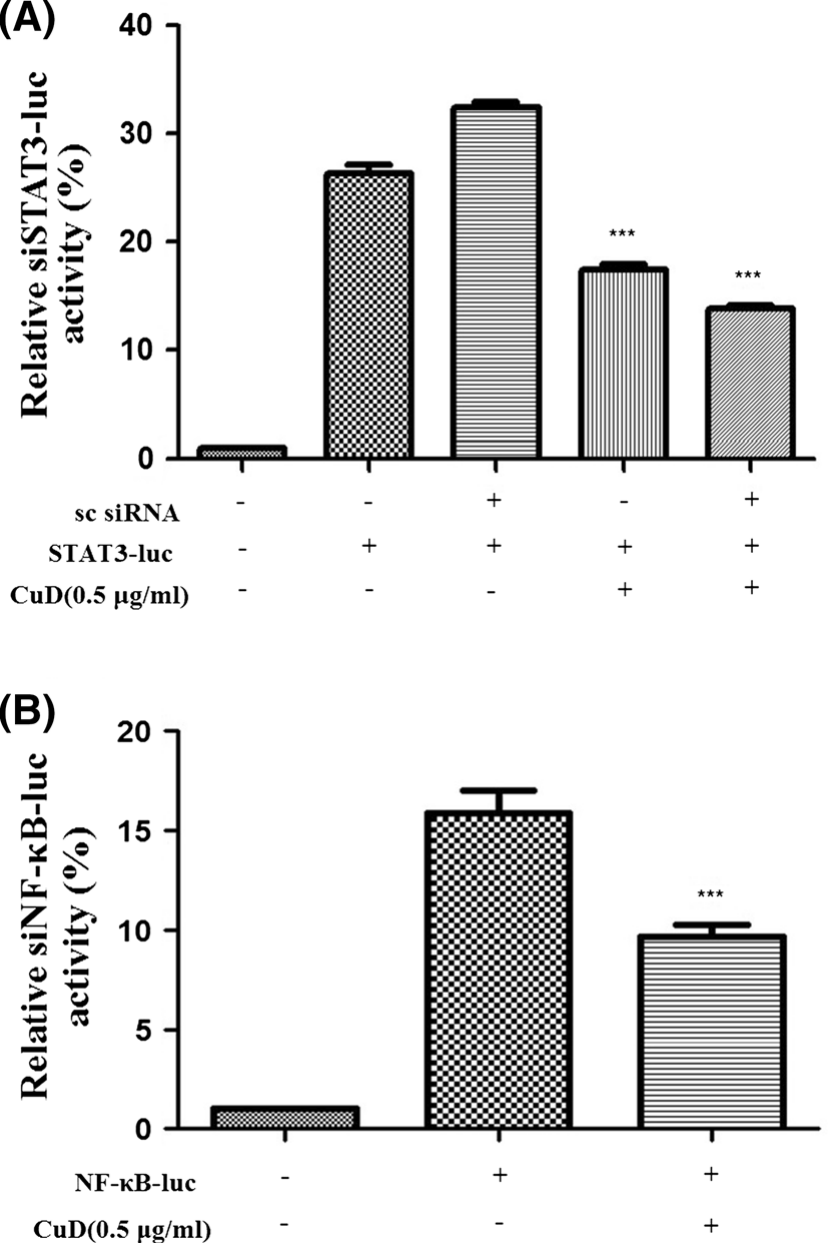
Cucurbitacin D inhibits Stat3 and NF-κB transcription in MCF7 cells. MCF7 cells were transfected with the indicated siRNA or plasmid, and then treated with each drug for 24 h. MCF7 cells were then treated with cucurbitacin D (0.5 μg/mL). Afterward, lysates were analyzed using the dual-luciferase reporter assay. Each value represents the mean ± SD. All data are *p* < 0.0001 by Student *t*-test

Although cucurbitacin D inhibits Stat3 and NF-κB translocation ability, it is not known whether the compound can regulate Stat3 and NF-κB transcriptional activity. Therefore, we performed luciferase reporter gene assays to detect Stat3 and NF-κB transcriptional activity. We found that cucurbitacin D significantly repressed STAT3 and NF-κB transcription activity in MCF7 cells (Fig. 5a, b).

### Cucurbitacin D induces apoptosis and G2/M cell cycle arrest in MCF7/ADR cells

**Fig. 6:**
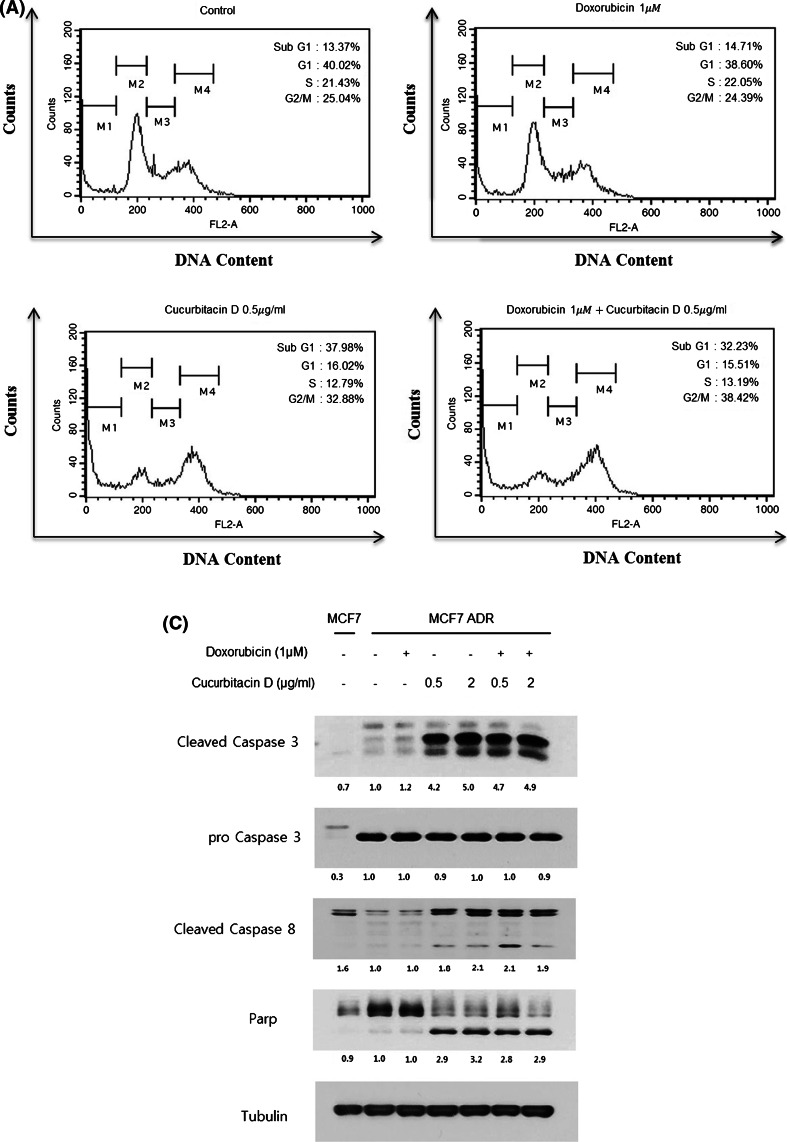

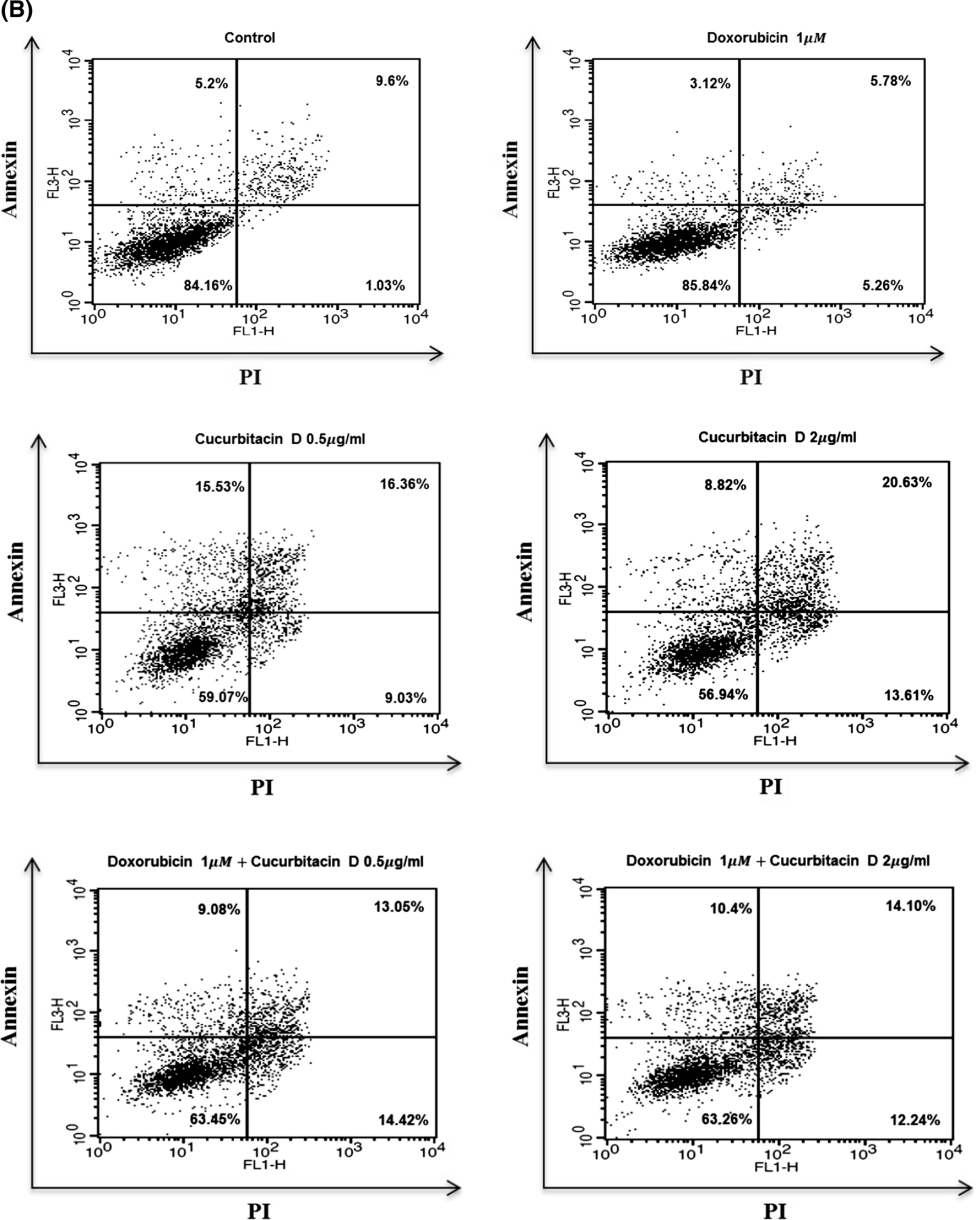
Cucurbitacin D induces G2/M cell cycle arrest and apoptosis in MCF7/ADR cells. MCF7/ADR cells were treated with cucurbitacin D (0.5 μg/mL) in the presence and absence of doxorubicin (1 μM) for 24 h. Cell cycle distribution was analyzed using a FACS flow cytometer (**a**). MCF7/ADR cells were treated with the indicated drugs for 24 h and subjected to Annexin V/PI assay. **b** Effect of cucurbitacin D on the expression of cleaved caspase-3, cleaved caspase-8, and cleaved PARP. MCF7/ADR cells were treated with cucurbitacin D (0.5 μg/mL) in the presence and absence of doxorubicin (1 μM) for 24 h. The cell lysates were subjected to Western blot analysis using specific antibodies (**c**)

To investigate whether cucurbitacin D inhibits cell proliferation by promoting changes in cell cycle progression, the effect of cucurbitacin D on the cell cycle profile was assessed using flow cytometry. For this purpose, MCF7/ADR cells were treated with cucurbitacin D (0.5 μg/mL) and/or doxorubicin (1 μM) for 24 h. The results demonstrated that cucurbitacin D induced an increase in the sub-G1 and G2/M populations in MCF7/ADR cells, suggesting that cucurbitacin D induces G2/M cell cycle arrest (Fig. 6a). We found that doxorubicin did not induce apoptosis in drug-resistant cells; however, cucurbitacin D treatment resulted in a 114 % increase in apoptosis (apoptotic cells were calculated after Annexin V/PI staining) when compared to that of control group. Doxorubicin with cucurbitacin D treatment resulted in a 145 % increase in apoptosis (apoptotic cells were calculated after Annexin V/PI staining) when compared to that of doxorubicin control group (Fig. 6b). Because the sub-G1 level was increased by cucurbitacin D treatment, we performed Annexin V/PI staining flow cytometry analysis to detect apoptotic cells. To confirm that caspase activation is involved in cucurbitacin D-induced apoptosis in MCF7/ADR cells, we found that cucurbitacin D up-regulated the levels of cleaved caspase-3, cleaved caspase-8, and cleaved PARP in MCF7/ADR cells (Fig. 6c).

## Discussion

In this study, we found that cucurbitacin D decreased cell proliferation and induced apoptosis by inhibiting Stat3 and NF-κB signaling in doxorubicin-resistant breast cancer cells, MCF7/ADR.

Among the Stat proteins, persistent activation of Stat3 is detected in human cancer cell lines and tumor tissues [24, 25]. These include breast cancer, lung cancer, pancreatic cancer, head and neck cancer, prostate cancer, ovarian cancer, melanoma, leukemias, and lymphomas. Stat3 activation in tumor cells is associated with cell proliferation, cell survival, invasion, angiogenesis, and metastasis [27]. Doxorubicin-treated cancer cells contained activated Stat3. Stat3 activation is linked to the development of doxorubicin resistance in cancer cell lines [28]. Stat3 is a predictive marker of drug resistance [9]. Therefore, we analyzed the expression and activation of Stat3 in MCF7 and doxorubicin-resistant MCF7/ADR cells. We also investigated whether Stat3 activation was associated with doxorubicin resistance in MCF7/ADR. We found that Stat3 was constitutively activated in MCF7/ADR, and this activation was disrupted by cucurbitacin D. This result suggests that cucurbitacin D inhibits cell growth and induces apoptosis by inhibiting Stat3 signaling. Additionally, cucurbitacin D inhibits nuclear translocation of Stat3, as revealed by immunocytochemistry.

NF-κB has an important effect on cell growth and inhibition of apoptosis. Activation of NF-κB promotes proliferation, inflammation, and tumorigenesis in cancer [12–14]. To investigate the mechanism by which cucurbitacin D decreases cell growth and induces apoptosis in MCF7/ADR cells, we made nuclear and cytosolic fractions and analyzed them by Western blot to measure the levels of NF-κB signaling molecules. We found that cucurbitacin D increased IκB level in the cytosol and suppressed the nuclear translocation of p-NF-κB, resulting in apoptosis. Cucurbitacin D suppressed cell growth in both MCF7 and MCF7/ADR cells in a dose- and time-dependent manner. This growth inhibition was accompanied by cell morphology changes and cell cycle arrest. The cell cycle is controlled by numerous mechanisms ensuring correct cell division. Additionally, cancer progression is related to aberrant cell cycle regulation [29]. G2/M DNA damage checkpoint consists of an arrest of the cell in G2 just before mitotic entry in response to genotoxic stress [30]. We found that cucurbitacin D increases the sub-G1 population and G2/M population in MCF7/ADR cells. To confirm apoptosis, we performed the Annexin V-FITC/PI assay and Western blot in MCF7/ADR cells. Cucurbitacin D treatment resulted in a 114 % increase in apoptosis when compared to that of control group. Additionally, results of Western blots demonstrate that cucurbitacin D up-regulated the levels of cleaved caspase-3, cleaved caspase-8, and cleaved PARP. Thereby, cucurbitacin D obviously induces apoptosis in MCF7/ADR cells. Because cucurbitacin D inhibits cell growth and induces apoptosis in doxorubicin-resistant breast cancer cells, MCF7/ADR, cucurbitacin D could be used as a useful compound to treat drug resistance. Targeting Stat3 and NF-κB may also be useful to treat breast cancer. Our study clearly demonstrates that cucurbitacin D overcomes doxorubicin resistance in breast cancer cells.

## Conclusions

These results clearly demonstrate that cucurbitacin D could be used as a useful compound to overcome doxorubicin resistance.
